# Supplementary material for: Two-timescale response of a large Antarctic ice shelf to climate change
Source: Nat Commun. 2021 Mar 31;12:1991. doi: 10.1038/s41467-021-22259-0 (PMC8012367; doi:10.1038/s41467-021-22259-0)
Supplement: Supplementary file 1 — Supplementary Information [file 41467_2021_22259_MOESM1_ESM.pdf]

Supplementary Information for "Two-timescale response of a large Antarctic ice shelf to climate change"

Kaitlin A. Naughten, Jan De Rydt
Sebastian H. R. Rosier, Adrian Jenkins
Paul R. Holland, Jeff K. Ridley

Supplementary Note 1: Coastal wind correction

As described in Methods, biases in the UKESM coastal winds used to force ÚaMITgcm meant that a correction scheme was required to obtain a realistic present-day simulation. Our correction scheme extends the work of *Mathiot et al.* [1], who calculated scaling factors for winds around the Antarctic coastline. These scaling factors, which varied in space but were constant in time, corrected coastal winds from a low-resolution atmospheric simulation before they were used to force an ocean-sea ice model.

Unlike *Mathiot et al.* [1], who scaled the magnitudes of the u and v components of the wind separately, we apply the correction in polar coordinates. This approach is more general, as it does not assume any particular orientation of the coastline. We scale the magnitude of the wind and rotate

the angle, such that the time-averaged quantities agree with reanalyses over the observational period. The scaling factors and rotation angles are held constant over all simulations.

First, we compare the years 1979-2014 between the UKESM historical simulation [2] (first ensemble member) and the ERA5 atmospheric reanalysis [3]. The wind fields from each product are time-averaged and linearly interpolated to the higher-resolution MITgcm grid. At each horizontal point within the domain, the ratio of wind speed between ERA5 and UKESM is calculated. This ratio is capped at 3, and is smoothed with a 2D Gaussian filter with a radius of 2 grid cells. The result is tapered to 1 over the first 150 km from the coast (land or ice front) following a cosine function. The final scaling factors are shown in Supplementary Figure 1a. Similarly, at each horizontal point, we calculate the difference in wind angle between ERA5 and UKESM. The angle is converted to the range $(-180°, 180°)$ and is tapered to 0 over the first 150 km from the coast as before. The result is shown in Supplementary Figure 1b.

At each timestep during the MITgcm simulation, the UKESM zonal and meridional winds (u, v) are converted to local polar coordinates $(r, \theta) = (\sqrt{u^2 + v^2}, \tan^{-1} v/u)$. The magnitude r is multiplied by the scaling factor (Supplementary Figure 1a) and the angle is added to the wind rotation angle (Supplementary Figure 1b) before converting back to longitude-latitude coordinates $(r \cos\theta, r \sin\theta)$.

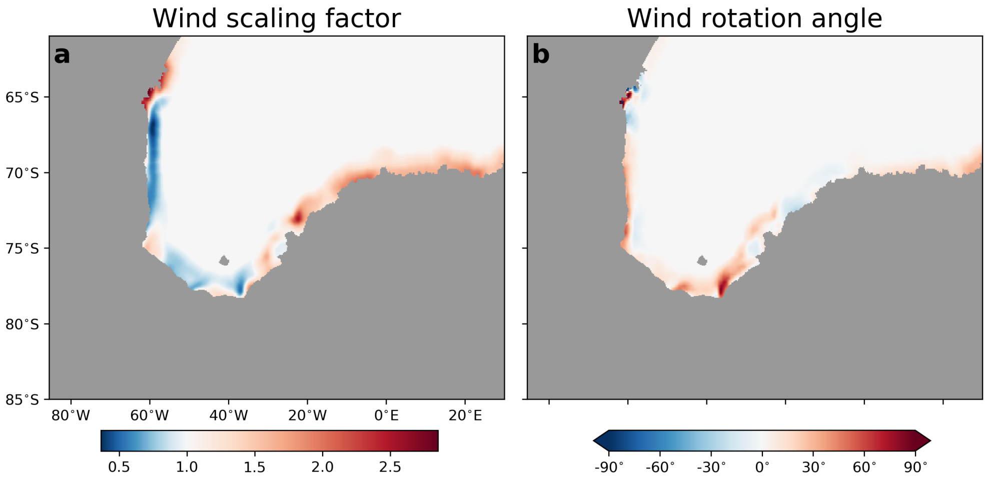

Supplementary Figure 1: Correction fields used for the UKESM coastal winds. (a) Scaling factor (dimensionless). (b) Rotation angle, in degrees.

Supplementary Note 2:

Comparison of present-day simulation with observations

The Weddell Sea ocean configuration underpinning ÚaMITgcm has previously been published [4] and found to perform well. However, these previous simulations were forced with atmospheric reanalyses, with ocean state estimates on the lateral boundaries. To ensure that the model is still reliable when forced with UKESM output, we conduct an additional ÚaMITgcm simulation over the observational period. This simulation is forced with UKESM's historical experiment over the years 1979-2014 [2] (first ensemble member), which differs from the pre-industrial baseline analysed in the main text [5]. It is initialised from the end of the spinup (Methods). The results

are comparable to simulations forced with the ERA5 reanalysis (not shown). This similarity indicates that the coastal wind correction described in Section is effective, and that additional biases in UKESM over this region are minimal or inconsequential.

Here, we compare the results with recent observations in the FRIS region, noting that such observations are sparse and uncertain, given the inaccessibility of the region and its high interannual variability. Also note that the UKESM historical simulation inevitably has a different pattern of internal climate variability to that which occurred in reality, which makes the comparison with observations imperfect. However, the 35-year simulation period is sufficient to average out shorter modes of variability such as the El Niño Southern Oscillation.

Simulated net basal mass loss from FRIS (68 Gt/y) agrees with the satellite estimates of *Moholdt et al.* [6] (58-190 Gt/y), *Joughin and Padman* [7] (59-108 Gt/y), and *Depoorter et al.* [8] (10-90 Gt/y), but is lower than the estimate of *Rignot et al.* [9] (110-200 Gt/y). The spatial pattern of basal melt rates also agrees with satellite estimates, as shown in Supplementary Figure 2 for *Moholdt et al.* [6]. The only significant region where the sign of the basal mass balance disagrees is in the northeast Filchner cavity, near the Bailey Ice Stream and Slessor Glacier (see Figure 7 of the main text for region labels). Here, the model simulates net melting rather than net freezing, which is caused by an intermittent bottom flow of Berkner Bank HSSW into

the Filchner Trough. This model bias is discussed by *Naughten et al.* [4] and could be related to an incomplete representation of fast ice in the model.

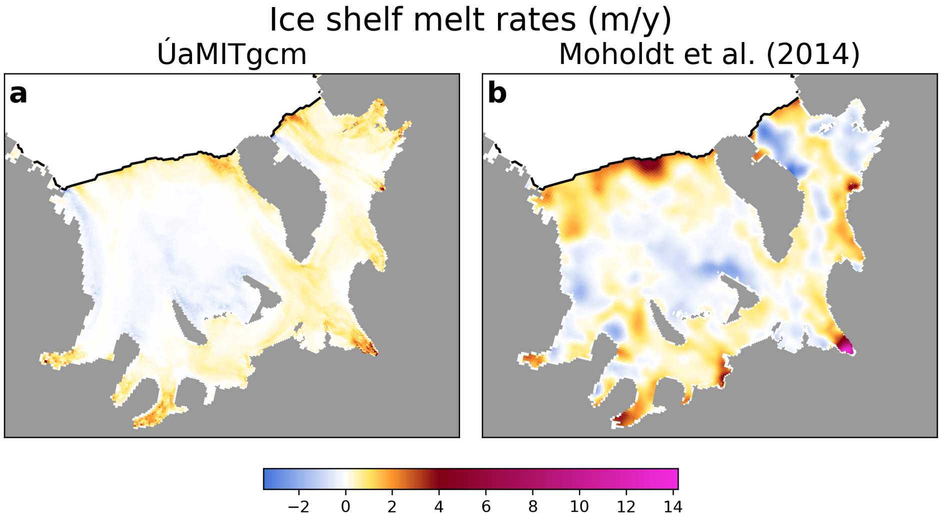

Supplementary Figure 2: Ice shelf basal melt rates in m/y. (a) Time-averaged output from the ÚaMITgcm historical simulation, from 1979-2014. (b) Satellite estimate of *Moholdt et al.* [6].

Although the spatial pattern is otherwise accurate, the overall amplitude of simulated melting and freezing is too low compared to the estimate of *Moholdt et al.* [6]. This behaviour is likely caused by the absence of tides [10], which cannot be explicitly simulated in this configuration on long timescales due to computational expense [4]. The lack of explicit tides does not affect the conclusions of this study, which rely on changing density gradients. The mechanism by which variability in density gradients leads to variability in melt rates is robust and has been identified in observations [11] despite the presence of tides, which increase mixing.

Upstream of FRIS are the ice shelves of the Eastern Weddell region. These ice shelves are not coupled to Úa, and therefore have fixed geometry, but their basal melt rates still evolve within MITgcm. Transient changes in meltwater from these ice shelves can impact salinity in front of FRIS, as discussed in the main text. The ice shelves with the largest contribution to meltwater are the adjoining Brunt and Riiser-Larsen Ice Shelves (BRLIS) and the adjoining Ekstrom, Jelbart, and Fimbul Ice Shelves (EJFIS). Averaged over the historical simulation, BRLIS has a simulated basal melt flux of 24.5 Gt/y, which is within the observational estimates of *Rignot et al.* [9]. The simulated melt flux from EJFIS is 56 Gt/y, somewhat above the range given by *Rignot et al.* (26.8 ± 14 Gt/y). Note that ice shelf meltwater from outside the ÚaMITgcm domain is implicitly taken into account, via the lateral boundary conditions from UKESM (Methods).

Next, simulated water mass characteristics in three key regions near the FRIS ice front are compared to CTD observations collected by the PS111 cruise in 2018 [12] (Supplementary Figure 3). The observed temperature and salinity profiles generally fall within the range of interannual variability simulated by ÚaMITgcm, with particularly good agreement over Berkner Bank (Supplementary Figure 3b). The main exception is ISW in the bottom of the Filchner Trough (Supplementary Figure 3c), which is too warm in the model by approximately 0.3°C, reflecting the inflow of Berkner Bank HSSW as discussed above. In the Ronne Depression (Supplementary Figure 3a),

observed bottom salinity is very slightly higher than the range of simulated profiles. This indicates that either 2018 had unusually high sea ice production in the Ronne Polynya, or the simulations have mean-state bias of slightly low sea ice production in this region (a possibility discussed by *Naughten et al.* [4]). The model also exhibits an overly shallow and sharp mixed layer in the Ronne Depression and Filchner Trough, with low surface salinities. However, this shallow mixed layer does not significantly affect the FRIS cavity, as most inflow happens at depth.

The FRIS region is not affected by ocean model drift, as demonstrated by the 150-year piControl simulation (see Figure 5 in the main text). There is no significant trend in southern Weddell Sea temperature or salinity, FRIS cavity salinity, or sea ice area. The only trends which are significant at the 95% level are extremely small and have low correlation coefficients: FRIS mass loss (trend of -0.04 Gt/y, $r^2 = 0.03$) and FRIS cavity temperature (trend of 8×10^{-5} °C/y, $r^2 = 0.08$).

Biases in the deep water masses inherited from UKESM are minimal. Compared to the World Ocean Atlas [13, 14], the UKESM's deep water masses have a temperature bias within ±0.3°C at the eastern boundary of MITgcm (Supplementary Figure 4), and ±0.5°C at the northern boundary (not shown). Deep salinity biases are negligible in both regions. Note that the MITgcm domain is more sensitive to conditions at the eastern boundary, due to the direction of the coastal current.

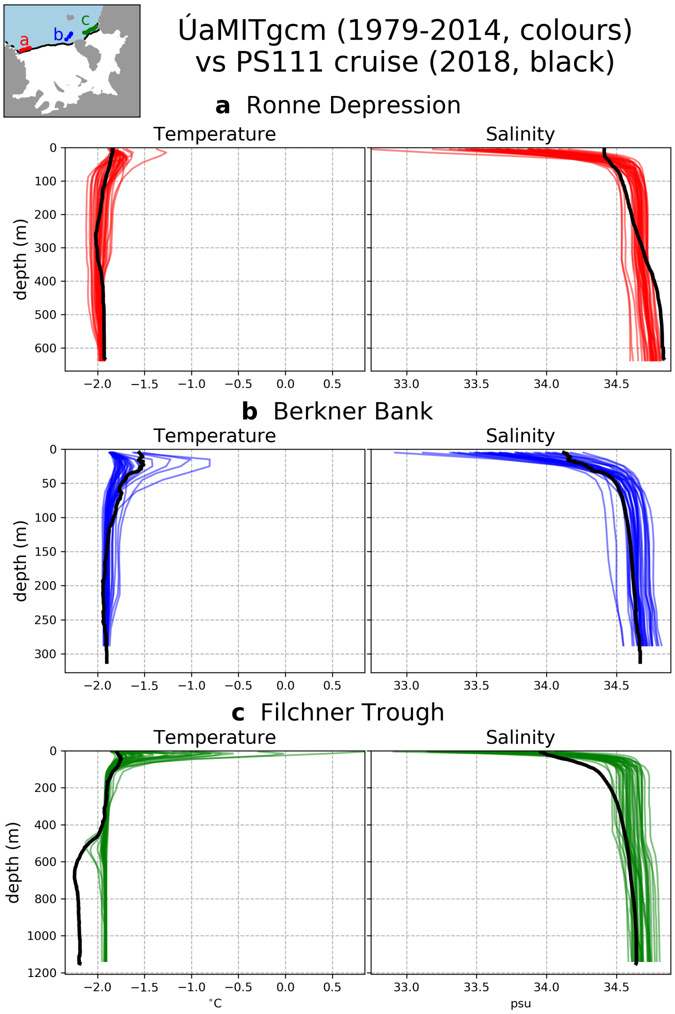

Supplementary Figure 3: Temperature and salinity profiles at key regions near the FRIS ice front. The thick black lines show CTD observations collected from January-March 2018 during the PS111 cruise [12] at the given locations in the inset map. The thin coloured lines show ÚaMITgcm output for each year of the historical simulation (1979-2014), averaged over January-March. For both model and observations, each profile is averaged over the points shown for the given region: (a) Ronne Depression (red points in inset map); (b) Berkner Bank (blue points); (c) Filchner Trough (green points).

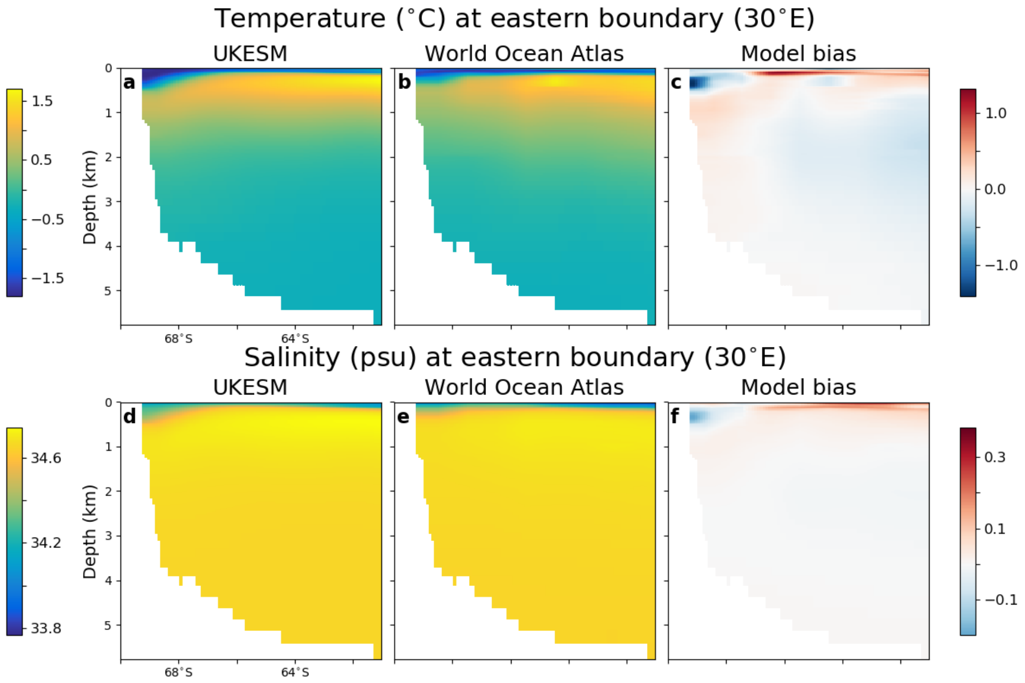

Supplementary Figure 4: (a-c) Temperature and (d-f) salinity at 30°E, the eastern boundary of MITgcm. (a, d) Output from the UKESM (averaged over years 1979-2014 of the historical simulation). (b, e) Observations from the the World Ocean Atlas [13, 14]. (c, f) Model bias (UKESM minus World Ocean Atlas). All fields are interpolated to the MITgcm grid.

Supplementary Note 3: Animations of cavity changes

Supplementary Movie 1 shows animations of bottom temperature and salinity in the FRIS cavity and on the surrounding continental shelf, during the abrupt-4xCO2 simulation and its 50-year extension. The video shows a marked freshening of the continental shelf, while freshening of the deep cavity is slower. The cavity cools, especially near the deep grounding lines, and in the outflowing ISW in Filchner Trough. From the beginning, there are minor

pulses of modified WDW into the eastern flank of Filchner Trough, but they do not reach the ice front in significant quantities until year 79, when Stage 2 begins. The first pulses into the cavity have a forked appearance because they occur at mid-depth, and the bottom of the central Filchner Trough is still filled with cold ISW. The warm pulses gradually become stronger and move further into the cavity as the ISW is eroded, eventually travelling around the back of the cavity to reach the central Ronne Ice Shelf.

Also note the modified WDW pulses into the Ronne Depression and across the central Ronne Ice Shelf front. These are existing flow pathways of WDW onto the continental shelf, but the heat is no longer fully extracted by sea ice formation.

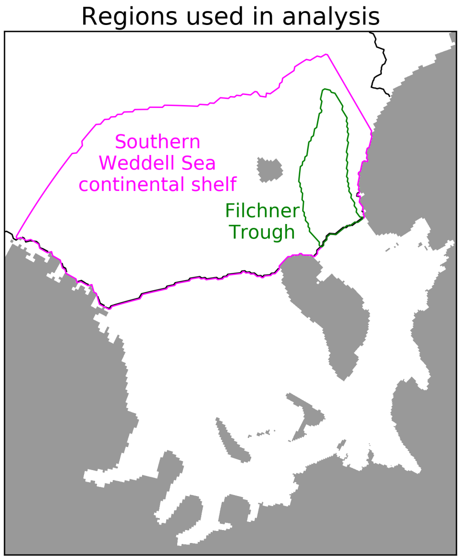

Supplementary Figure 5: Regions used for analysis in this manuscript, contoured and labelled by colour. The Southern Weddell Sea continental shelf (magenta) is defined by the bounds 70-30°W, 79-72°S, the 1250 m isobath, and the ice front. The Filchner Trough (green) is defined by the bounds 40-35°W, 79-75°S, the 650 m and 1250 m isobaths, and the ice front.

Supplementary References

[1] Mathiot, P., B. Barnier, H. Gallée, J. M. Molines, J. L. Sommer, M. Juza, and T. Penduff, Introducing katabatic winds in global ERA40 fields to simulate their impacts on the Southern Ocean and sea-ice, *Ocean Modelling*, *35*, 146–160, doi:10.1016/j.ocemod.2010.07.001 (2010).

[2] Met Office Hadley Centre, WCRP CMIP6: Met Office Hadley Centre (MOHC) UKESM1-0-LL model output for the "historical" experiment, https://catalogue.ceda.ac.uk/uuid/59c10ac7bea2424f8eb64f0e310a2d4f (2019).

[3] Copernicus Climate Change Service, ERA5: Fifth generation of ECMWF atmospheric reanalyses of the global climate, https://www.ecmwf.int/en/forecasts/datasets/reanalysis-datasets/era5 (2017).

[4] Naughten, K. A., A. Jenkins, P. R. Holland, R. I. Mugford, K. W. Nicholls, and D. R. Munday, Modelling the influence of the Weddell Polynya on the Filchner-Ronne Ice Shelf cavity, *Journal of Climate*, *32*, 5289–5303, doi:10.1175/JCLI-D-19-0203.1 (2019).

[5] Eyring, V., S. Bony, G. A. Meehl, C. A. Senior, B. Stevens, R. J. Stouffer, and K. E. Taylor, Overview of the Coupled Model Intercomparison Project Phase 6 (CMIP6) experimental design and organization, *Geoscientific Model Development*, *9*, 1937–1958, doi:10.5194/gmd-9-1937-2016 (2016).

[6] Moholdt, G., L. Padman, and H. A. Fricker, Basal mass budget of Ross and Filchner-Ronne ice shelves, Antarctica, derived from Lagrangian analysis of ICESat altimetry, *Journal of Geophysical Research: Earth Surface*, *119*, 2361–2380, doi:10.1002/2014JF003171 (2014).

[7] Joughin, I., and L. Padman, Melting and freezing beneath Filchner-Ronne Ice Shelf, Antarctica, *Geophysical Research Letters*, *30*, 1477, doi:10.1029/2003GL016941 (2003).

[8] Depoorter, M. A., J. L. Bamber, J. A. Griggs, J. T. M. Lenaerts, S. R. M.

Ligtenberg, M. R. van den Broeke, and G. Moholdt, Calving fluxes and basal melt rates of Antarctic ice shelves, *Nature*, *502*, 89–92, doi: 10.1038/nature12567 (2013).

[9] Rignot, E. J., S. S. Jacobs, J. Mouginot, and B. Scheuchl, Ice-Shelf Melting Around Antarctica, *Science*, *341*, 266–270, doi:10.1126/science.1235798 (2013).

[10] Hausmann, U., J. B. Sallée, N. C. Jourdain, P. Mathiot, C. Rousset, G. Madec, J. Deshayes, and T. Hattermann, The Role of Tides in Ocean-Ice Shelf Interactions in the Southwestern Weddell Sea, *Journal of Geophysical Research: Oceans*, *125*, e2019JC015847, doi:10.1029/2019JC015847 (2020).

[11] Nicholls, K. W., and S. Østerhus, Interannual variability and ventilation timescales in the ocean cavity beneath Filchner-Ronne Ice Shelf, Antarctica, *Journal of Geophysical Research*, *109*, C04014, doi: 10.1029/2003JC002149 (2004).

[12] Janout, M. A., H. H. Hellmer, T. Hattermann, O. Huhn, J. Sültenfuss, S. Østerhus, L. Stulic, S. Ryan, M. Schröder, and T. Kanzow, FRIS revisited in 2018: On the circulation and water masses at the Filchner and Ronne Ice Shelves in the southern Weddell Sea, *Journal of Geophysical Research: Oceans*, *in review* (2020).

[13] Locarnini, R. A., A. V. Mishonov, J. I. Antonov, T. P. Boyer, H. E.

Garcia, O. K. Baranova, M. M. Zweng, C. R. Paver, J. R. Reagan, D. R. Johnson, M. Hamilton, and D. Seidov, World Ocean Atlas 2013. Vol. 1: Temperature., *NOAA Atlas NESDIS 81*, https://www.ncei.noaa.gov/data/oceans/woa/WOA18/DOC/woa18_vol1.pdf (2013).

[14] Zweng, M. M., J. R. Reagan, J. I. Antonov, A. V. Mishonov, T. P. Boyer, H. E. Garcia, O. K. Baranova, D. R. Johnson, D. Seidov, and M. M. Bidlle, World Ocean Atlas 2013, Volume 2: Salinity, *NOAA Atlas NESDIS 82*, https://www.ncei.noaa.gov/data/oceans/woa/WOA18/DOC/woa18_vol2.pdf (2013).
