# Supplementary material for: Two-timescale response of a large Antarctic ice shelf to climate change
Source: Nat Commun. 2021 Mar 31;12:1991. doi: 10.1038/s41467-021-22259-0 (PMC8012367; doi:10.1038/s41467-021-22259-0)
Supplement: Supplementary file 4 — Description of additional supplementary files [file 41467_2021_22259_MOESM4_ESM.docx]

Description of additional supplementary information:

Title: Supplementary Movie 1

Description: Animation of bottom temperature (°C) and salinity (psu) in the Filchner-Ronne Ice Shelf cavity and surrounding continental shelf, during every month of the abrupt-4xCO2 simulation and its 50-year extension.
